# 3D Modelling of Mass Transfer into Bio-Composite

**DOI:** 10.3390/polym13142257

**Published:** 2021-07-09

**Authors:** Marouane Kabbej, Valérie Guillard, Hélène Angellier-Coussy, Caroline Wolf, Nathalie Gontard, Sébastien Gaucel

**Affiliations:** IATE, Univ Montpellier, CIRAD, INRAE, Institut Agro, 34060 Montpellier, France; marouane.kabbej@umontpellier.fr (M.K.); helene.coussy@umontpellier.fr (H.A.-C.); wolf.caroline1@gmail.com (C.W.); nathalie.gontard@inrae.fr (N.G.); sebastien.gaucel@inrae.fr (S.G.)

**Keywords:** 3D numerical modelling, three-phase model, interphase, Finite Element Method, water vapor permeability, composite

## Abstract

A three-dimensional model structure that allows considering interphase layer around permeable inclusions is developed to predict water vapor permeability in composite materials made of a matrix Poly(3-HydroxyButyrate-*co*-3-HydroxyValerate) (PHBV) including Wheat Straw Fiber (WSF) particles. About 500 two-phase structures corresponding to composites of different particles volume fractions (5.14−11.4−19.52 % v/v) generated using experimental particles’ size distribution have permitted to capture all the variability of the experimental material. These structures have served as a basis to create three-phase structures including interphase zone of altered polymer property surrounding each particle. Finite Element Method (FEM) applied on these structures has permitted to calculate the relative permeability (ratio between composite and neat matrix permeability $P/P_{m}$). The numerical results of the two-phase model are consistent with the experimental data for volume fraction lower than 11.4 %v/v but the large upturn of the experimental relative permeability for highest volume fraction is not well represented by the two-phase model. Among hypothesis made to explain model’s deviation, the presence of an interphase with its own transfer properties is numerically tested: numerical exploration made with the three-phase model proves that an interphase of 5 µm thick, with diffusivity of $D_{i}\geq 1\times 10^{-10}\;m^{2}\cdot s^{-1},$ would explain the large upturn of permeability at high volume fraction.

## 1. Introduction

In the last 10 years, a serious interest has been devoted to the modelling of mass transfer properties of composite materials and more particularly of gas and vapor permeability. Permeability is a key functional property that determine the functionality of a given material and thus its application (membranes, packaging, etc.) [1,2]. Predictive modelling of such properties is thus of primary importance to design new tailored materials corresponding to the application requirements. Among innovative materials, composites obtained with the addition of at least two non-miscible constituents with different properties, offer new promising possibilities in terms of applications because of the synergy between components that creates unique macroscopic properties that would not be achievable otherwise from the individual constituents [3,4]. Incorporation of particles in a continuous phase as polymer matrix permits to significantly modulate the mass transfer properties of the resulting composite and makes this strategy very attractive for different fields of applications (barrier materials, membrane gas separation, etc.).

A lot of studies were carried out on the modeling of mass transfer properties in (nano/micro) composites containing either impermeable particles for the application of barrier materials as reviewed, for example, by Cui et al. (2015) or Zid et al. (2018) [5,6] or permeable particles in the field of separation membranes [7]. In the following of this state of the art, we will focus principally on references dealing with mass transfer modelling in system where permeable particles are dispersed in a continuous polymer matrix.

Historically, first models developed were analytical relationship for estimation of the overall macroscopic diffusivity or permeability of a composite from structural parameters (volume fraction, aspect ratio, etc.) and mass transfer properties of the individual component [8,9,10]. Based on analogies with electrics and thermo-mechanic theories, models based upon Maxwell, Bruggeman, Pal or Lewis-Nielsen were proposed [10,11]. Developed for regular dispersion of homogeneous-size particles of idealized geometry (e.g., spheres), they were applied with more or less success to predict permeability of binary systems, mostly in the field of mixed-matrix membranes [7,12]. Their main drawbacks were lack of representativity of the real 3D structure of the composite, assumption of spherical shape of filler which renders the particle geometry insignificant [13], a validity limit restricted to low filler volume fraction (diluted system) [7] and necessity to have access to permeability of the disperse particles which is not always easy to accurately determine [14]. In order to improve their reliability, efforts have been made to complexify the analytical models to consider, for instance, more complex particles geometry such as oblong prolate particles instead of spheres [13,15] or to extend range of validity to high particles volume fraction [16].

In parallel, to overcome the limitations of analytical approaches, some authors have proposed numerical models that attempt to consider the real 3D structure of the composite system and that solve the mass transfer using generally, Finite Element Method (FEM). We can cite, as recent examples in that field, the work of Monsalve-Bravo et al. [17] who model the three-dimensional (3-D) transport problem in full-scale mixed-matrix membranes using FEM or the work of Sharifzadeh and co-authors (2019) who simulated gas diffusion behavior in 3D composite filled with permeable spherical particles randomly dispersed [18]. The two aforementioned studies confirmed that the permeability is positively correlated to filler volume fraction and particle size. However, they did not consider the heterogeneity of the particle size, which is often the case in real bio-composites.

At the opposite of simplifications made in sphere-based models, some authors tried to consider directly the true internal 3D microstructure as Jiang and co-authors (2020) who simulated with a 3D FEM modelling approach, water diffusion in jute/PLA composite using a real structure observed by X-ray tomography and mass transfer properties of the individual components [19]. The numerical results were in good agreement with experimental measurements. Nevertheless, since the simulations were only performed on a unique and small volume of material observed by X-ray tomography, the representativeness of the macroscopic diffusion in the material in its wholeness were questionable. 

In the previous cited works whether analytical or numerical ones, the presence of an interphase at the particle/matrix interface which exhibit mass transfer properties differing substantially from those of the bulk matrix is always neglected. Role of this interphase is of paramount importance which can lead to a percolating interphase network inside of the composite, as highlighted by Qiao and Brinson (2009) [20] or Petsi and Burganos (2012) [21] in mixed-matrix membranes and by Zid et al. [22] on nanocomposites with impermeable fillers. Aforementioned studies of Qiao and Brinson and Petsi and Burganos proposed a two-dimensional numerical model to study the impact of interphase zones on the overall properties of the composites (mechanical properties for Qiao and Brinson and effective permeability for Petsi and Burganos). In their study, Zid et al. proposed 3D finite element model to predict mass diffusion in (nano) composites but in their case, particles, with ideal ordered distribution, did not participate to the overall diffusion. Some attempts have also been made to upgrade analytical approaches considering three-phase media—see among others the work of Petropoulos et al. (2015) [23]—with a double binary formula application that consider first particles with interphase (surrounding zones) as pseudo-particles of effective permeability P_E_ dispersed in the bulk matrix. P_E_ is identified using a standard analytical formula for binary medium. Same standard analytical formula is then applied to the virtual binary composite containing the pseudo particles. To eliminate limitations imposed by the sphere equations that physically limits the maximal volume fraction investigated (random packing of congruent spheres imposed a non-negligible lattice volume), change in particle shape from spherical to cubic was proposed and found not significant, at least in the polymer-gas permeability area [16]. However, this approach is restricted to regular dispersion of homogenous particle-size and could not take into account heterogeneity in interphase thickness, particles size and distribution into the matrix. 

The objective of the present paper is to propose a three-dimension, three-phase numerical model to compute effective permeability of bio-composite structures where particles are permeable and widely contributed to the overall mass transport. This model aims at deciphering the role of each phase contributing to the overall transport (matrix, inclusion) and, in particular, the role of the third phase (interphase). To go beyond state of the art, and compensate weaknesses of previous numerical and analytical approaches, numerical computations of the effective permeability are carried out on model structures stemming from experimental observation of the composite—poly(3-hydroxybutyrate-*co*-3-hydroxyvalerate) (PHBV) as continuous phase and wheat straw fibers (WSF) as dispersed phase—where particles, idealized as spheroids, are randomly distributed in a representative volume element (RVE). This approach allows to represent all the complexity of the real bio-composite while simplifying it to keep sufficiently low computation time which makes possible exploration of a lot of structures and filler volume fractions. Existence of an interphase layer around the inclusions is considered: interphase layer was built from the two-phase structure by allowing this layer to freely overlap with interphase layers around neighboring particles. The effect of the diffusivity and thickness of the interphase layer on the effective permeability calculated is discussed, as well as relevance of diffusivity values chosen for individual components (matrix and particle). Conclusions of the present work is of paramount importance in the field of material science where modelling of permeability in composite material considering at the same time 3D structure, permeable particles and presence of an interphase and percolating interphase network for high filler fraction was never considered before.

## 2. Materials and Methods

### 2.1. Experimental Parameters for Water Vapor Transfer

To instantiate and validate the numerical models, a real case study of water vapor transfer into a composite consisting of Poly(3-HydroxyButyrate-*co*-3-HydroxyValerate) (PHBV) matrix and wheat straw fiber (WSF) particles was considered, based on previous experimental investigations performed in our laboratory. Some experimental outputs coming from this previous work [24] were used as input parameters for the numerical model. These inputs are explained below and recalled in Table 1.

- Effective moisture diffusivity $D\;\left[m^{2}\cdot s^{-1}\right]$ value of each phase matrix and particle.
- The boundary concentrations of water vapor $\left[\mathrm{mol}\cdot m^{-3}\right]$ in the PHBV matrix in contact with dry air and with humid air (relative humidity of 95%). These concentrations were determined using the experimental water vapor sorption isotherm of PHBV film at 20 °C.
- Water vapor partition coefficient K=54.52, calculated as the slope of the linear relation between the water concentration in PHBV matrix and WSF particles, obtained from experimental water vapor sorption isotherm at 20 °C for matrix and particles.

**Table 1:** Summary of the experimental parameters for water vapor transfer used in this work.

Sample	Diffusivity ^a^ $\times 10^{-12}$ $\left[m^{2}\cdot s^{-1}\right]$	Permeability $\times 10^{-13}$ $\left[\mathrm{mol}\cdot m\cdot m^{-2}\cdot s^{-1}\cdot Pa^{-1}\right]$	Upper Boundary Concentration ^a^ $\left[\mathrm{mol}\cdot m^{-3}\right]$	Lower Boundary Concentration ^a^ $\left[\mathrm{mol}\cdot m^{-3}\right]$	Partition Coefficient ^a^
PHBV matrix	2.615±0.56	8.29±3.96 ^b^	337.14	0	54.52
WSF particle	18.39±4.93	1664±451 ^c^	-	-	

^a^ Obtained from dynamic sorption experiments (DVS, Dynamical Vapor Sorption system, Surface Measurement System, London, UK) [24]; ^b^ Correspond to the average of three experimental sets of measures made in the same laboratory and directly measured from gravimetric experiment (Modified ASTM procedure) [24,25,26]. ^c^ Calculated as the product of experimental particle diffusivity by experimental particle solubility obtained from dynamic sorption experiments (DVS, Dynamical Vapor Sorption system, Surface Measurement System, London, UK) [24].

### 2.2. 2D Image Analysis

Image processing and analyzing was performed on 2D mosaic images of 2869 Wheat Straw Fibre (WSF) particles by using MATLAB (images provided by Wolf and Berthet from co-authors’ laboratory [27,28]). Real particles were assimilated to ellipses whose shape descriptors (major $a_{f}$ and minor $b_{f}$ axis) were measured.

### 2.3. 3D Structure Generation

The MATLAB code developed for generating our 3D structures was based on Tschopp MATLAB code [29]. The code generates 3D microstructures composed of a population of non-overlapping ellipsoid particles heterogeneously distributed in size and orientation, within a periodic RVE.

### 2.4. Mathematical Modelling and Geometry

#### 2.4.1. 3D Structure Generation

**Figure 1:**
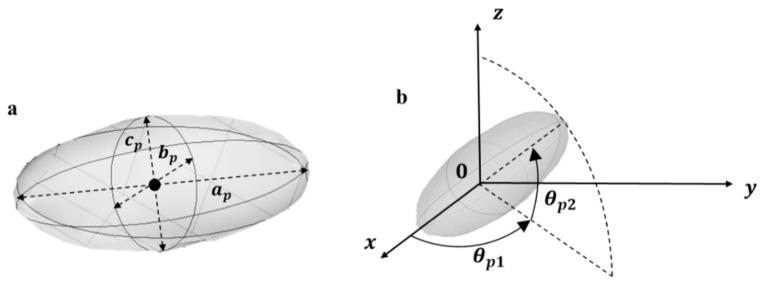
Modelling a particle as a spheroid in 3D space. (**a**) $a_{p}$: major axis, $b_{p}$: minor axis, $c_{p}$: third axis (**b**) $\theta_{p1}$: Azimuth angle. $\theta_{p2}$: elevation angle.

Two–phase system. The two-phase composite structure is generated in a cuboid shaped representative volume element (RVE) defined by $\left(x,y,z\right)\;\in \;\left[0,L_{x}\right]\times [0,L_{y}]\times [0,L_{z}]$, where *z* is the overall diffusion direction. The RVE is supposed periodic along its vertical faces, to represent an infinite repetitive structure along x and y axis. In the present work, particles are considered as elongated spheroids, i.e., ellipsoids of revolution over the first (major) axis $a_{p}$, where the second axis $b_{p}$ and the third axis $c_{p}$ are equal and lower than the major axis (Figure 1).

Structure generation required the RVE size and the particles volume fraction $\varphi_{p}$ as inputs. First, geometric parameters of particles (major axis $a_{p}$ and aspect ratio $\alpha_{p}=a_{p}/b_{p}$) are randomly (non-uniform distribution) generated until the target volume fraction of particles is reached, using the stop criterion $\left|\varphi_{p,target}-\varphi_{p}\right|\leq 0.01\%v/v$. Second, these particles are sequentially positioned in the periodic RVE, by decreasing first axis following the steps below.

- Step 1. The position (center coordinates $x_{p},y_{p},z_{p}$) and orientation (azimuth $\theta_{p1}$ and elevation $\theta_{p2}$ angle) are randomly drawn using uniform distributions.
- Step 2. The non-overlapping of the particle with the horizontal faces of the RVE (z=0 and $z=L_{z}$) and with the existing particles is tested.

If the non-overlapping tests were successful, the particle was added to the structure and then the next particle is considered for tests 1–2. If at least one non-overlapping condition was not satisfied, then a new position was drawn, orientation being unchanged, and step 2 was performed again on the updated particle. This last sequence was repeated until the particle was added to the structure. 

**Figure 2:**
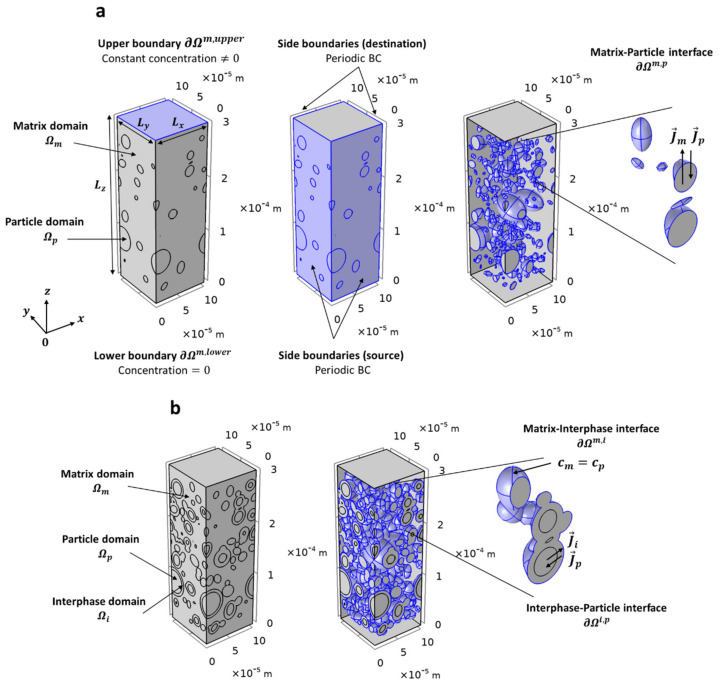
Representation of the RVE showing: (**a**) the boundaries conditions at the six composite faces and at the interface matrix–particle for a two–phase structure; (**b**) the boundaries conditions at the matrix–interphase and interphase–particle interfaces for a three–phase structure.

It should be noted that if a particle intercepted one of the vertical faces of the RVE then, the particle section outside from the RVE was shifted to the opposite face, in order to ensure the periodicity of the RVE (Figure 2). Organizational chart summarizing the structure generation algorithm could be found in Supplementary Materials (Figure S1).

Three–phase system. The three-phase composite structure was built by adding an interphase of controlled but fixed thickness around each particle of the already generated two-phase structures. The interphase volume was thus different for each particle keeping constant the initial particle volume. Three different interphase thicknesses $e_{i}$ were considered $e_{i}=1-2.5-5\;µm$.

#### 2.4.2. Governing Equations

Mass transfer in the micro-composite is described by Fick’s second law of diffusion in both the matrix $\Omega_{m}$ and the particle $\Omega_{p}$ domains for the two-phase system (Figure 2a) and also in the interphase domain $\Omega_{i}$ for the three-phase system (Figure 2b). In stationary regime and in the absence of mass source, this law is expressed by the following partial differential:

(1) $$div\left({\vec{J}}_{k}\right)=0$$

where

(2) $${\vec{J}}_{k}\left(x,y,z\right)=-D_{k}\vec{\nabla }c_{k}\left(x,y,z\right)\;\mathrm{in}\;{\vec{J}}_{k}\left(x,y,z\right)=-D_{k}\vec{\nabla }c_{k}\left(x,y,z\right)$$

where $D_{m}$, $D_{p}$ and $D_{i}$ $\left[m^{2}\cdot s^{-1}\right]$ are the diffusivity coefficient in the matrix, the particle and the interphase domains respectively and they are considered constant (not concentration/temperature/time dependent). *k* stands for the domain considered, matrix (m), particle (*p*) or interphase (*i*). ${\vec{J}}_{m}$, ${\vec{J}}_{p}$ and ${\vec{J}}_{i}$ are thus the molar surface flux vector $\left[\mathrm{mol}\cdot m^{-2}\cdot s^{-1}\right]$ depending on $c_{m},$ $c_{p}$ and $c_{i}$ $\left[\mathrm{mol}\cdot m^{-3}\right]$ that are the concentration of the water vapor in the matrix, the particle and the interphase domain respectively.

#### 2.4.3. Boundary Conditions

Periodic boundary conditions were imposed on the vertical side boundaries of the RVE, which consist to impose equality of concentration and flux on the so-called (source) and (destination) boundaries (Figure 2). The periodic boundary conditions allowed simulating an infinite repetitive structure. Constant concentrations (*cte*) were imposed on the upper face $\partial \Omega^{m,upper}$ and the lower face $\partial \Omega^{m,lower}$ of the matrix (Equations (3) and (4) and Figure 2a). Particle and interphase domains are assumed to not overlap the upper and lower faces of the RVE.

(3) $$c_{m}=cte\neq 0\;\mathrm{at}\;\partial \Omega^{m,upper}$$

(4) $$c_{m}=0\;\mathrm{at}\;\partial \Omega^{m,lower}$$

Two-phase system. There are discontinuities in the concentration profile $c_{m}\neq c_{p}$ at the matrix-particle interface $\partial \Omega^{m,p}$. In that respect, matrix and particle concentrations are considered linearly dependent at the matrix-particle interface $\partial \Omega^{m,p}$ by the dimensionless partition coefficient $K=c_{p}/c_{m}$. To get continuous flux at the matrix-particle interface $\partial \Omega^{m,p}$, a special type of boundary condition using the stiff-spring method [30] was applied:

(5) $${\left(-\vec{J}\cdot \vec{n}\right)}_{m}=M\left(c_{p}-K\cdot c_{m}\right)\;\mathrm{at}\;\partial \Omega^{m/p}$$

(6) $${\left(-\vec{J}\cdot \vec{n}\right)}_{p}=M\left(K\cdot c_{m}-c_{p}\right)\;\mathrm{at}\;\partial \Omega^{p/m}$$

where *M* is a (non-physical) velocity $\left[m\cdot s^{-1}\right]$ large enough to let the concentration differences in the brackets approach zero, thereby satisfying $K=c_{p}/c_{m}$. This boundary condition gives a continuous flux across the interfaces provided that *M* is sufficiently large. In all simulations *M* was taken equal to 1000 $m\cdot s^{-1}$.

Three-phase system. The interphase was considered to have the same sorption properties than the matrix, continuity of concentration at the matrix-interphase interface $\partial \Omega^{m,i}$ was applied:

(7) $$c_{m}=c_{i}\;\mathrm{at}\;\partial \Omega^{m,i}$$

At the interphase–particle interface $\partial \Omega^{i,p}$, the same condition than previously applied for the two-phase system was adopted with partition coefficient $K=c_{p}/c_{i}$. The boundary conditions using the stiff-spring method in the three-phase system are thus:

(8) $${\left(-\vec{J}\cdot \vec{n}\right)}_{i}=M\left(c_{p}-K\cdot c_{i}\right)\;\mathrm{at}\;\partial \Omega^{i/p}$$

(9) $${\left(-\vec{J}\cdot \vec{n}\right)}_{p}=M\left(K\cdot c_{i}-c_{p}\right)\;\mathrm{at}\;\partial \Omega^{p/i}$$

where *M* is the same than above. 

#### 2.4.4. Effective Permeability Evaluation

The solution of the boundary value problem yielded the molar concentration field $c_{i}\left(x,y,z\right)\;\left[\mathrm{mol}\cdot m^{-3}\cdot s^{-1}\right]$ and the molar surface flux vector $\vec{J}\left(x,y,z\right)$ $\left[\mathrm{mol}\cdot m^{-2}\cdot s^{-1}\right]$ of the permeating specie at the discretization points of each domain $\Omega_{k}\;\left(k=m,p,i\right)$. The molar flux $\phi_{z}$ $\left[\mathrm{mol}\cdot s^{-1}\right]$ (along *z*-axis) across the upper $\left(z=L_{z}\right)$, middle $\left(z=L_{z}/2\right)$ and lower $\left(z=0\right)$ cross sections was calculated by:

(10) $$\phi_{z}\left(z\right)=\int_{0}^{L_{x}}\int_{0}^{L_{y}}\;J_{z}\left(x,y,z\right)dxdy\;$$

where $J_{z}$ is the molar surface flux (along *z*-axis) at the discretization points. Then, the effective permeability $\left[\mathrm{mol}\cdot m\cdot m^{-2}\cdot s^{-1}\cdot {\mathrm{Pa}}^{-1}\right]$ of the micro-composite was finally given by:

(11) $$P=\frac{\phi_{z}}{L_{x}\times L_{y}}\cdot \left(\frac{L_{z}-0}{p_{upper}-p_{lower}}\right)$$

where $L_{x}\times L_{y}$ is the surface of the faces $\left[m^{2}\right]$, $L_{z}$ is the RVE thickness $\left[m\right]$, $p_{upper}$ and $p_{lower}$ are the water vapor pressure $\left[\mathrm{Pa}\right]$ imposed on the upper $\left(z=L_{z}\right)$ and the lower $\left(z=0\right)$ faces of the RVE respectively and $\phi_{z}$ is the molar flux $\left[\mathrm{mol}\cdot s^{-1}\right]$.

### 2.5. Numerical Simulations

The 3D boundary value problem of diffusion was solved by using the numerical Finite Element Method (FEM) using COMSOL Multiphysics 5.5 software. An unstructured mesh consisting of tetrahedral elements was used for the discretization of the composite geometry.

For simulation, the Transport of Diluted Species physics interface of the Chemical Reaction Engineering module and the COMSOL CAD Import module, were used. The simulations were performed in DELL computer with Intel Xeon E-2176M Processor (2.7 GHz) and 32 Gb of Ram. 

The entire computational procedure (from structure generation to simulation) was driven within the MATLAB environment and was partially automated via the COMSOL LiveLink for MATLAB module (from the step of importing geometry data into COMSOL).

## 3. Results and Discussion

The 3D modelling approach presented above was applied to the prediction of the water vapor permeability into bio-composite materials made of PHBV as continuous phase and WSF as dispersed and permeable phase. Experimental data corresponded to the average of three experimental sets of measures made in the same laboratory for three increasing volume fractions $\left(\varphi_{p}=5.14-11.4-19.52\;\%v/v\right)$ [24,25,26]. For the sake of clarity, in the following, the results are presented and discussed in terms of relative permeability, i.e., the ratio between the composite permeability and that of the neat matrix $\left(P/P_{m}\right)$. Experimental results showed an increase of the relative permeability with increasing fiber particles volume fraction due to the hydrophilic nature of particles which are much more permeable than the PHBV matrix considered [24].

Preliminary calculations made using the analytical solution the most representative of the system studied here, i.e., the Maxwell–Wagner–Sillar equation [13] for dilute dispersion of ellipsoids, confirmed that the system was much more complex than a simple binary composite. Indeed, the sharp upturn of the experimental curve for the highest volume fraction (19.52 %*v*/*v*) was not captured by the analytical approaches (see Figure S2 in Supplementary Materials). Among hypotheses usually made explaining such a discrepancy, presence of an interphase is the most frequently quoted [20,21]. A three-phase numerical model was thus developed to explore the role of this interphase on the effective macroscopic permeability of the composite. To meet this objective, the strategy applied was first to build the two-phase model and then to add an interphase layer around the inclusions of the two-phase structures to obtain three-phase systems. In order to generate simplified but representative 3D structures, the first step was to determine the particle morphology, size and shape distributions of particles in the experimental composite.

### 3.1. From Particle Morphology to 3D Structure Generation

**Figure 3:**
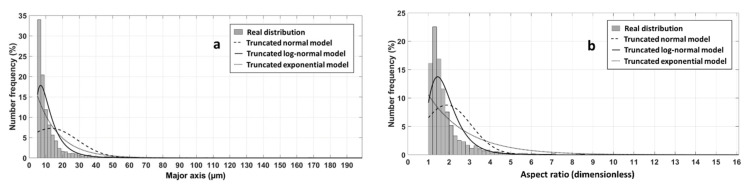
Comparison between experimental distribution and fitted distributions of (**a**) major axis and (**b**) aspect ratio obtained from 2869 particles.

2D images containing a total number of 3594 WSF particles were analyzed to obtain geometrical descriptors of the particle morphology. Each particle was described by the parameters of its inertia ellipse, major axis $a_{f}$ and aspect ratio $\alpha_{p}$ (ratio of major axis to minor axis $\alpha_{p}=a_{p}/b_{p}$), as it allowed to replace the actual particle by an ellipse while preserving centroid, area and orientation. It is worth noting that the very fine WSF particles $\left(a_{f}<5\;µm\right)$ were discarded, reducing the total number of particles from 3594 to 2869. Indeed, these very fine particles represent a negligible volume fraction, about 0.118% (volume calculated considering real fibers as spheroids) and their contribution to mass transfer is negligible. Moreover, for numerical simulations, the absence of very fine particles allows to avoid using extremely fine meshes that increase the computation time. The empirical distributions (number frequency) of major axis and aspect ratio of the final set of particles showed monomodal shape (Figure 3) with mean E=13.15 µm and standard deviation SD=15.75 µm for the major axis, E=1.9 and SD=1.17 for the aspect ratio, respectively. 

The distributions of these shape descriptors were fitted with three theoretical distribution laws, truncated normal, truncated log-normal and truncated exponential laws (Figure 3). Figure 3 shows that the fitted truncated log-normal distribution is the closest to the real distribution. The identified mean and standard deviation of the truncated log-normal distribution after fitting were E=13.4 µm and SD=8.14 µm for the major axis, E=1.97 and SD=0.75 for the aspect ratio. The expressions of the mean and standard deviation of the truncated log-normal distribution are given in Appendix A. Experimental means of both major axis and aspect ratio are well described by the truncated log-normal distribution while standard deviations are a bit underestimated. This latter fact is explained by the presence of a very few number of long particles which are difficult to capture with continuous distributions.

**Figure 4:**
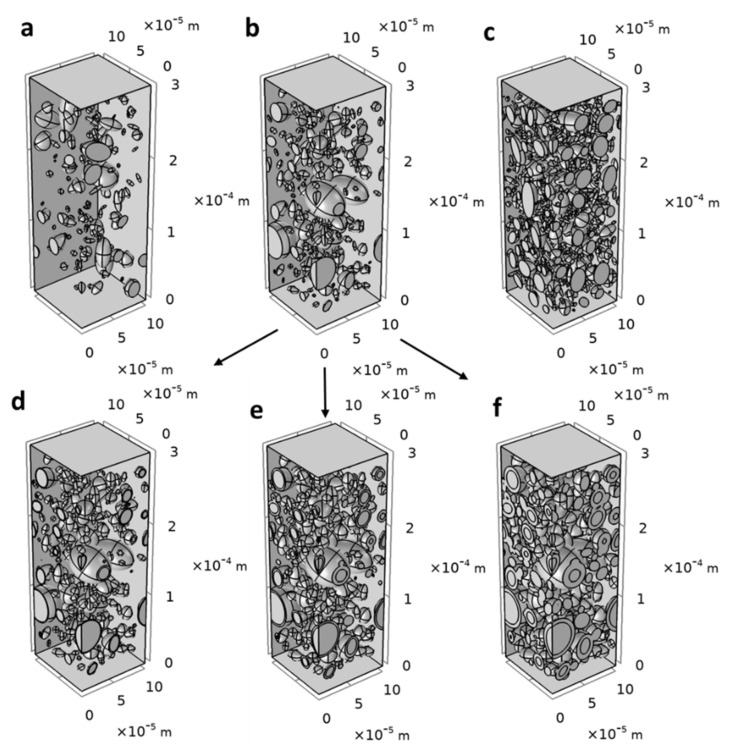
Examples of 3D composite structures (two-phase system) generated for particles volume fraction corresponding to true composite materials (**a**) $\varphi_{p}=5.14\;\%v/v$ (122 particles), (**b**) $\varphi_{p}=11.4\;\%v/v$ (214 particles) and (**c**) $\varphi_{p}=19.52\;\%v/v$ (611 particles). Examples of 3D composite structures (three-phase system) built by adding interphase to the two-phase structure (**b**) with different interphase thicknesses (**d**) $e_{i}=1\;µm,$ (**e**) $e_{i}=2.5\;µm$ and (**f**) $e_{i}=5\;µm$.

In order to generate 3D structures, the particles were modelled as elongated spheroids, i.e., ellipsoids of revolution along the first axis, the longer one. Major axis $a_{f}$ and aspect ratio $\alpha_{p}$ were randomly generated according to the truncated log-normal distributions. In absence of 3D experimental assessment of the particle morphology, assumption of spheroid shape allowed to mimic elongated particles, contrarily to spheres, while keeping smooth boundary which ease the meshing and the numerical solving, contrarily to cylinders or ribbons. No constraints were added for the orientation of particles. The method used (see Section 2) allowed to generate hundreds of composite structures, in moderate time, with particle volume fraction ranging from 0 to 38 %v/v (equivalent to the mass fraction of 48 %wt). Figure 4 illustrates some structures obtained with volume fractions equal to experimental ones $\left(\varphi_{p}=5.14-11.4-19.52\;\%v/v\right)$.

This approach based on extracting information from real images to generate size distribution was rarely used in the field of mass transfer study, since in most of numerical studies in this field, the generated size distributions are parameterized arbitrarily as in [31,32].

### 3.2. Simulations of Two-Phase Model

#### 3.2.1. Selection of Mesh and RVE Sizes

**Figure 5:**
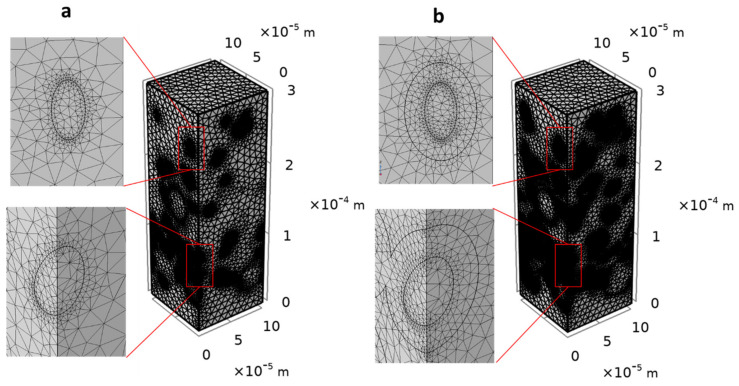
Tetrahedral mesh presentation of 3D composite structures corresponding to particles volume fraction of $\varphi_{p}=11.4\;\%v/v$ (240 particles): (**a**) two–phase system and (**b**) three–phase system.

First, the influence of the mesh size on the numerical accuracy of the solution was studied. Preliminary simulations performed on a hundred structures of RVE size of $100\times 100\times 300\;{µm}^{3}$ for each composite ($\varphi_{p}=5.14-11.4-19.52\;\%v/v$) revealed that the best convergence of solutions was obtained with the mesh element size of $\left[0.45-10.5\right]\;µm$ (predefined in COMSOL) which was then applied in all the further simulations. Indeed, the relative permeability stopped varying approximatively from the mesh element size of $\left[0.45-10.5\right]\;µm$ (see Figures S3–S5 in Supplementary Materials). Generally, the mesh was more refined in the regions that require a higher resolution, such as near the matrix-particle interface (Figure 5a) or the matrix-interphase and the interphase-particle interface (Figure 5b).

Then, influence of the RVE size was investigated. For the present study, fixed RVE thickness $\left(L_{z}=300\;µm\right)$ was imposed which was consistent with the thickness of the material represented. It is indeed important to represent the water flux on the entire thickness of the material to capture all the complexity of the system in the direction of the monodirectional flow (with side effects on the upper and lower faces where particles did not outcrop as experimentally observed); therefore, $L_{z}=300\;µm$ was kept as constant value. Further simulations performed on about 100 structures for each RVE size $\left(100\times 100\times 300;\;150\times 150\times 300;\;200\times 200\times 300{\;µm}^{3}\right)$ and each particle volume fraction ($\varphi_{p}=5.14-11.4-19.52\;\%v/v$) revealed that the RVE size did not significantly impact the results of mass flux and permeability calculation. For example, for 19.52 %v/v the permeability $\left[\mathrm{mol}\cdot m\cdot m^{-2}\cdot s^{-1}\cdot {\mathrm{Pa}}^{-1}\right]$ found were $7.87\pm 0.29\times 10^{-13}$ for a RVE of dimensions $100\times 100\times 300\;{µm}^{3}$, $7.80\pm 0.21\times 10^{-13}$ for 150×150×300 ${µm}^{3}$ and $7.88\pm 0.21\times 10^{-13}$ for $200\times 200\times 300\;{µm}^{3}$. Therefore, a minimal RVE of $\left(100\times 100\times 300\;{µm}^{3}\right)$ was chosen for all further simulations (Figure 4), permitting to achieve reasonable computational time.

In total, no fewer than 500 two-phase structures of RVE size of (100 × 100 × 300 µm^3^) were generated using experimentally observed particle size distribution: 185 structures for $\varphi_{p}$ = 5.14 %*v*/*v*, 199 structures for $\varphi_{p}$ = 11.4 %*v*/*v* and 158 structures for $\varphi_{p}$ = 19.52 %*v*/*v*.

#### 3.2.2. Selection of the Number of Structures to Analyze

For each filler load, the variability of numerical vapor permeability was analyzed, depending on the number of structures considered. As expected, accumulation of structures led to a stabilization of the mean permeability and a decrease of its standard deviation. Regarding the numerical variability obtained on water vapor permeability, it was found that numerical standard deviation became acceptable (i.e., less than 10%) for accumulation of results obtained on at least 10 structures, whatever the filler load. In the following, permeability was computed for the whole set of structures generated in order to completely ensure stable results and also to capture all the numerical variability and get rid of potential outliers caused by minority structures. By multiplying the number of computed structures, the variability of the material structure could be integrated more easily, leading to a better characterization and prediction of the material properties compared to works relying on actual 3D structures, e.g., costly 3D tomographic pictures [19].

#### 3.2.3. Numerical Results of the 2-Phase Model

**Figure 6:**
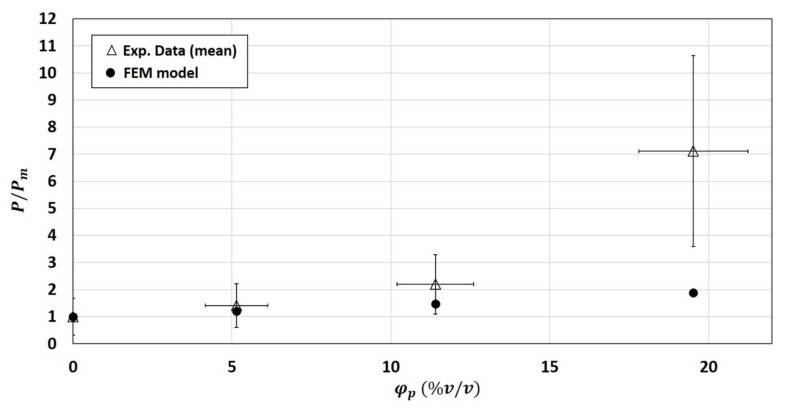
Comparison between experimental and numerical relative permeability $P/P_{m}$ (calculated using the 2-phase model).

As expected, the numerical permeability values obtained for the 2-phase system were not able to capture the upturn of permeability ratio for high volume fraction, even if good prediction of the of the experimental relative permeability ratio were obtained for the composites of $\varphi_{p}=5.14\;\%v/v$ and $\varphi_{p}=11.4\;\%v/v$ (Figure 6). 

Three different hypotheses could be made to explain this deviation:

1. the physical properties of the fiber particle and especially its diffusivity value would be modified once embedded into the polymer matrix compare to the one measure on the native component,
2. the diffusivity of the polymer matrix, measured before fiber particles addition, would be modified after fiber particles addition and therefore not well representative of what occurs in the composite material,
3. the presence of an interphase, third compartment with its own physical properties, at the interface matrix/particle would influence the overall permeability into the composite.

All these hypotheses were tested in the following, especially on the composite of $\varphi_{p}=19.52\;\%v/v$ where largest deviations were noted.

#### 3.2.4. Modification of Particles Diffusivity Values in the Two-Phase Model

**Figure 7:**
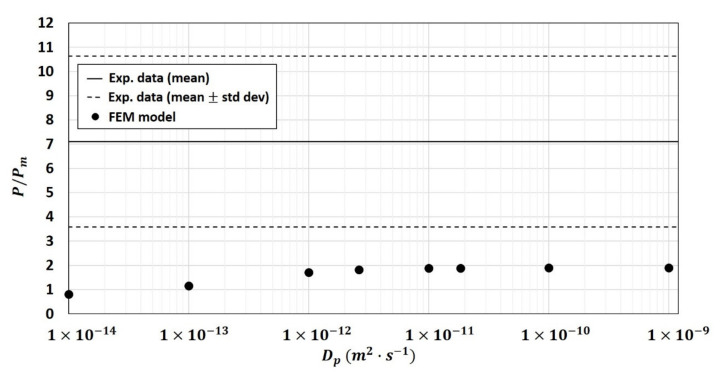
Effect of particle diffusivity $D_{p}$ on numerical relative permeability $P/P_{m}$ (calculated using the 2-phase model) into the composite of $\varphi_{p}=19.52\;\%v/v$ (hypothesis tested on 10 structures).

**Figure 8:**
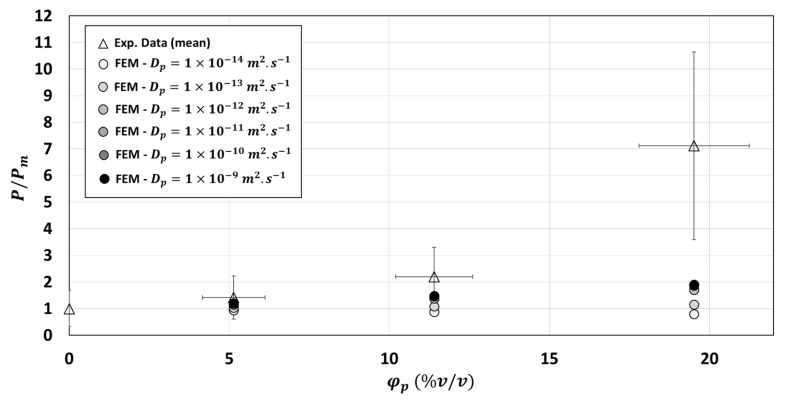
Comparison between experimental and numerical relative permeability $P/P_{m}$ (calculated using the 2-phase model) for different particle diffusivity values varying from $D_{p}=1\times 10^{-14}\;m^{2}\cdot s^{-1}$ to $D_{p}=1\times 10^{-9}\;m^{2}\cdot s^{-1}$ (hypothesis tested on 10 structures).

The first hypothesis tested in the 2-phase model was the modification of the diffusivity of the fiber particles $D_{p}$ once embedded in the polymer matrix. Figure 7, obtained on composite ($\varphi_{p}=19.52\;\%v/v$), showed that the relative permeability increased when $D_{p}$ increased from $1\times 10^{-14}\;m^{2}\cdot s^{-1}$ to $1\times 10^{-11}\;m^{2}\cdot s^{-1}$ and reached a plateau when $D_{p}\geq 1\times 10^{-11}\;m^{2}\cdot s^{-1}$. However, this increase was not enough to bring the numerical results closer to the experimental data for the composite of $\varphi_{p}=19.52\;\%v/v$ (Figure 8). 

In other words, modification of the diffusivity of the fiber particles $D_{p}$ in the two-phase model was not a valid hypothesis to explain deviation of the 2-phase model.

#### 3.2.5. Modification of Matrix Diffusivity Values in the Two-Phase Model

**Figure 9:**
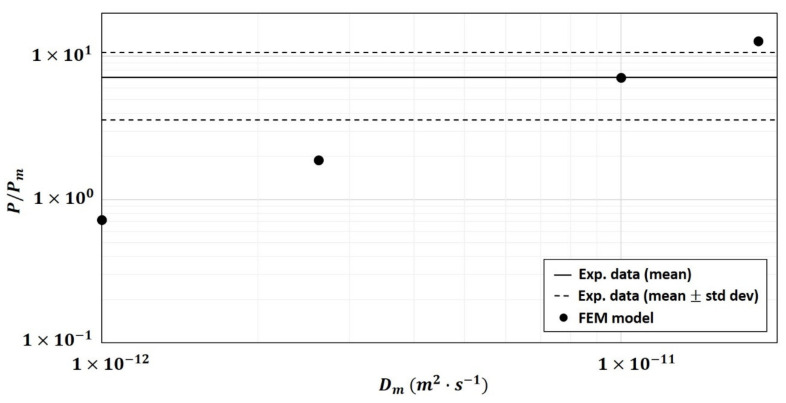
Effect of matrix diffusivity $D_{m}$ on numerical relative permeability $P/P_{m}$ (calculated using the 2-phase model) into the composite of $\varphi_{p}=19.52\;\%v/v$ (hypothesis tested on 10 structures).

**Figure 10:**
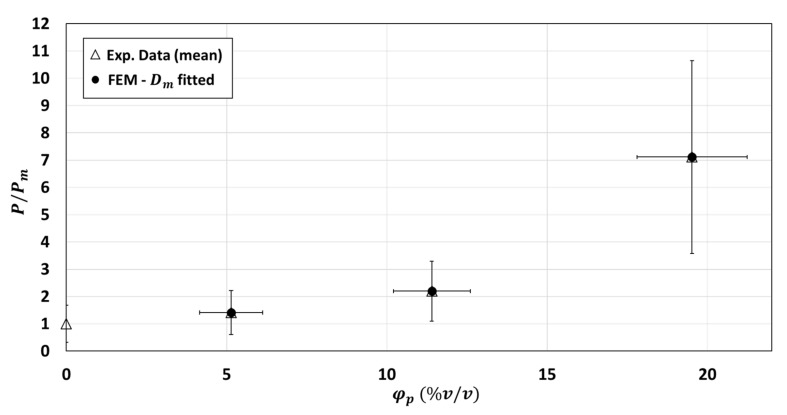
Comparison between experimental and numerical relative permeability $P/P_{m}$ (calculated using the 2-phase model): adjustment of numerical model to experimental data by fitting the matrix diffusivity $D_{m}$ (fitting performed on 10 structures for each composite).

The second hypothesis tested in the 2-phase model was the modification of diffusivity of the polymer matrix induced by fiber particles addition. As shown in Figure 9, the numerical relative permeability $P/P_{m}$ sharply increased when $D_{m}$ increased. The two-phase numerical model is highly sensitive to matrix diffusivity $D_{m}$. The optimal matrix diffusivity, i.e., the one leading to the best fit of the experimental data, was $D_{m}=3.08\times 10^{-12}\;m^{2}\cdot s^{-1}$ for $\varphi_{p}=5.14\;\%v/v$, $D_{m}=3.92\times 10^{-12}\;m^{2}\cdot s^{-1}$ for $\varphi_{p}=11.4\;\%v/v$ and $D_{m}=10.07\times 10^{-12}\;m^{2}\cdot s^{-1}$ for $\varphi_{p}=19.52\;\%v/v$ (Figure 10). These values of the diffusivity were, as expected, close to the experimental one for $\varphi_{p}=5.14\;\%v/v$ and 11.4 %v/v ($D_{m}=2.615\times 10^{-12}\;m^{2}\cdot s^{-1}$), as the 2-phase model relatively well predicted the experimental relative permeability for these two particle loads. On the contrary, the composite of $\varphi_{p}=19.52\;\%v/v$ would require a matrix diffusivity about three times higher than that currently used in the 2-phase model. That means that the experimental relative permeability could be well represented by the two-phase model for $\varphi_{p}=19.52\;\%v/v$ when $D_{m}$ is multiplied by 3. Relevance of this increase by three orders of magnitude in the polymer matrix is questionable but could be justified by modification of polymer crystallinity when high volume fraction of particles is added in the neat matrix. Impact of crystallinity on diffusivity of small-molecule penetrants in semicrystalline polymer is something well described in the literature [33] even if never confirmed for PHBV polymeric matrix. In the specific case of the PHBV matrix under study in the present work, Berthet et al. (2015) [26] measured by WAXD a decrease of crystallinity from 68.1 to 46.8%, between neat matrix and composite containing 20 %w/w (e.g., 11.4 %v/v) of WSF (same particle and same polymer than in the present work). This decrease of crystallinity rate means an increase of amorphous zones and higher mobility of the polymer in the continuous phase, which is generally ascribed to higher diffusivity values. For example, Trifol et al. (2020) [34] measured a water vapor diffusivity value multiplied by 2 between amorphous PLA and semicrystalline PLA with 35% of crystallinity. Considering the same effect (decrease of 35% of crystallinity rate leads to $D_{m}$ multiplied by 2) for our PHBV/WSF composite ($\varphi_{p}=11.4\;\%v/v$) would lead to a $D_{m}$ multiplied by 1.5. Extrapolating this to the $\varphi_{p}=19.52\;\%v/v$ composite, supposing that same drop of crystallinity rate would be observed between $\varphi_{p}=11.4\;\%v/v$ and $\varphi_{p}=19.52\;\%v/v$ than between $\varphi_{p}=11.4\;\%v/v$ and neat matrix, would lead to a $D_{m}$ value multiplied by 3. The hypothesis of strong modification of diffusivity of the polymer matrix induced by fiber particles addition is thus completely credible. Berthet et al. (2015) [26] also noted that in parallel to the decrease of crystallinity, addition of fibers induced a decrease of polymer molecular weight (219,340 to 169,906 g mol^−1^), which is also in favor of higher polymer mobility (less entanglements) and thus increased of $D_{m}$ value in composite. A modification of the crystal size was also highlighted by the authors: the size of crystals was increased from 1.03 to 1.18 nm in the presence of WSF. This higher crystal size is also in favor of an increase of diffusivity in the continuous phase because of lower tortuosity in the less tight crystal structure.

To sum up, hypothesis of a modification of diffusivity of the polymer matrix induced by fiber particles addition for high filler fraction would be realistic (for $\varphi_{p}=19.52\;\%v/v$ it is necessary to multiply $D_{m}$ by 3) and could be explained by a concomitant decrease of crystallinity rate, a decrease of polymer molecular weight and an increase of crystal size in the PHBV/WSF composite. 

It is however difficult to decipher the individual role of each effect (crystal size, molecular weight, crystallinity rate), all the more that increase of crystal size in composite hinder fiber/matrix adhesion [26] and would lead to interfacial phenomenon such as creation of an interphase with its own transfer properties at the interface particle/matrix. It is thus particularly relevant to investigate in the following the role of this interphase.

### 3.3. Simulations of Three-Phase Model

To build the three-phase model, an interphase of controlled but fixed thickness was added around each particle of the two-phase structures previously generated, reflecting a fixed degree of lack of adherence between inclusions and polymer. Interphase layers were allowed freely to overlap with interphase layers around neighboring particles. Three different thicknesses were explored: $e_{i}=1-2.5-5\;µm$, 5 µm being a worst-case scenario following literature analysis on that topic. Indeed, relevant thickness range of 0–2 µm was found in similar composite systems [35]. Also, a micro-thermal analysis on a glass fiber/epoxy composite revealed the presence of a zone of 4 µm thickness around the glass fibers with higher molecular mobility that is generally ascribed to higher diffusivity values [36]. 

**Figure 11:**
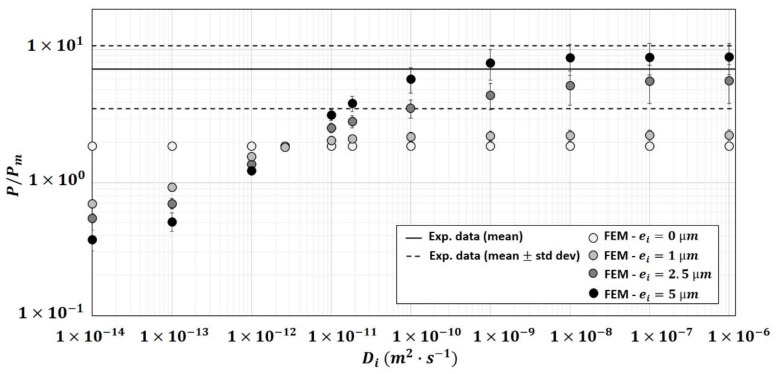
Evolution of the numerical relative permeability of the composite $\left(\varphi_{p}=19.52\;\%v/v\right)$ as a function of the diffusivity of interphase and for different thicknesses of the interphase The numerical results (symbols) correspond to the average of the relative permeability of 14 structures $\left(e_{i}=1\;µm\right)$, 19 structures $\left(e_{i}=2.5\;µm\right)$ and 6 structures $\left(e_{i}=5\;µm\right)$. Lines represent the experimental relative permeability for $\varphi_{p}=19.52\;\%v/v$: solid line for mean value, dashed lines for mean ± standard deviation.

A total of 69 structures for $\left(\varphi_{p}=5.14\;\%v/v\right)$, 93 structures for $\left(\varphi_{p}=11.4\;\%v/v\right)$ and 39 structures for $\left(\varphi_{p}=19.52\;\%v/v\right)$ were used for simulations (see Figure 4d–f for some examples). Simulations of the three-phase model were performed with several modalities for the diffusivity $D_{i}$ of the interphase and its thickness $e_{i}$. it must be noted that the standard deviation on the calculated relative permeability increases as the thickness and diffusivity of the interphase increase (Figure 11): this could be ascribed to the fact that less structures were analyzed for high $\varphi_{p}$ and high $\varphi_{i}$ values. Indeed, numerical constraints on structures with high $\varphi_{p}$ and high $\varphi_{i}$ have limited the number of structures analyzed and thus the number of permeability results considered. The equivalent graphs of Figure 11 for $\varphi_{p}=5.14\;\%v/v$ and $\varphi_{p}=11.4\;\%v/v$ were given in the Supplementary Materials (Figures S6 and S7).

Figure 11 showed that the numerical relative permeability is positively correlated to $D_{i}$ and $e_{i}$. Compared to the 2-phase results ($e_{i}=0$), the presence of an interphase can lead to an increase of the relative permeability, for $D_{i}>D_{m}$, or a decrease of the relative permeability, for $D_{i}<D_{m}$. For a thin interface, $e_{i}=1\;µm$, the diffusivity of the interface, $D_{i}$, had a strong effect on the relative permeability for $D_{i}<D_{m}$ but very limited impacts for $D_{i}>D_{m}$. For larger thicknesses ($e_{i}=2.5-5\;µm$), the results exhibit a greater impact of $D_{i}$ on the relative permeability for both $D_{i}<D_{m}$ and $D_{i}>D_{m}$. 

The increase of $e_{i}$ led, as expected, to an increase of the volume fraction of the interphase and so to an increase (resp. decrease) of the water vapor flux for $D_{i}>D_{m}$ (resp. $D_{i}<D_{m}$), e.g., for $\varphi_{p}=19.52\;\%v/v$, the volume fraction of the interphase was $\varphi_{i}=5.58\pm 1.37\;\%v/v$ for $e_{i}=1\;µm$, $\varphi_{i}=16.03\;\pm 3.2\;\%v/v$ for $e_{i}=2.5\;µm$ and $\varphi_{i}=29.24\;\pm 5.49\;\%v/v$ for $e_{i}=5\;µm$. This volume fraction, $\varphi_{i}$, can become equivalent or even higher than that of the particles, $\varphi_{i}\geq \varphi_{p}$. Even if the relationship between volume fraction of the interface and relative permeability is not straightforward (Figures S8–S10 in Supplementary Materials), the general trend observed is globally an increase of relative permeability for $D_{i}>D_{m}$ when the interphase volume fraction increased. For high volume fraction of the interphase, the interphases are strongly connected and some continuous pathways appeared along the main diffusion axis (z axis), which can act as percolation pathways and have an additional increasing impact on the mass transfer.

This typical impact of the presence of interphases on mass transfer within composites has already been observed by Zid et al. [22] for nanocomposite with impermeable fillers and by Petsi and Burganos [21] in mixed matrix membranes. Zid et al. [22] noticed that the interphase layers, depending on their diffusivity (weakly or highly diffusive) can be either beneficial or totally detrimental to the nanocomposite overall barrier properties. They particularly highlighted the impact of the effect of continuous diffusion paths, which may occur between overlapping interphases, that are particularly critical for the barrier performance in the case of highly diffusive interphases. This is perfectly in line with what has been observed for high filler volume fraction in the present work. Petsi and Burganos [21] went even further in their conclusions obtained on mixed matrix membranes by investigating the impact of constant interphase thickness (hypothesis made in the present work) or thickness that is proportional to the particle size. They concluded from the numerical exploration of their system that when the interphase thickness remains constant during particle decrease, the permeability increased thanks to the increased role of the interphase layer. It may be anticipated from this result that role of interphase layer would be higher in the case of composite with smaller particles. 

Indeed, a more detailed analysis of the results showed that the number of particles but also the spatial distribution impacted the relative permeability (Figures S8–S10 in Supplementary Materials). Indeed, as the interphase thickness was considered constant for all the particles, the total volume of the interphase is related to the dimensions of the spheroid particles, and to the position of particles as intersection of interphases can occur. Consequently, two different structures having the same particle volume fraction but different number of particles or different particle spatial distributions can lead to significantly different interphase volume fractions or interphase shape, and so different impacts on relative permeabilities, e.g., for $\varphi_{p}=19.52\;\%v/v$ and $e_{i}=2.5\;µm$: (i) $n_{p}=255$, $\varphi_{i}=15.42\;\%v/v$ and (ii) $n_{p}=253$, $\varphi_{i}=13.75\;\%v/v$ (Figure S9).

Figure 11 revealed that, for $\varphi_{p}=19.52\;\%v/v$, the three-phase model with thickness of $e_{i}=1\;µm$ was not sufficient to bring the numerical results closer to the experimental data whatever the $D_{i}$ value investigated between $1\times 10^{-14}$ and $1\times 10^{-6}\;m^{2}\cdot s^{-1}$. Good fit of the three-phase model on experimental relative permeability for this composite could be achieved only with thicker interphase (either $e_{i}=2.5\;µm$ or $e_{i}=5\;µm$) and by considering interphase diffusivity $D_{i}\geq 1\times 10^{-9}\;m^{2}\cdot s^{-1}$ for 2.5 µm of interphase thickness and $D_{i}\geq 1\times 10^{-10}\;m^{2}\cdot s^{-1}$ for 5 µm of interphase thickness. According to these results, the interphase would be as diffusive as or even more diffusive than the particles $D_{i}\geq D_{p}$.

**Figure 12:**
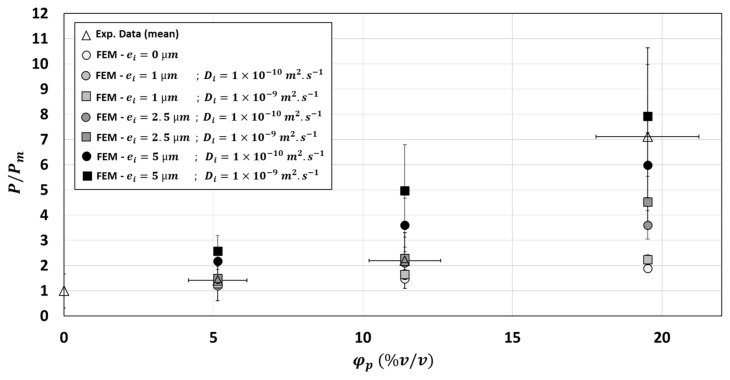
Comparison between experimental and numerical relative permeability: Effect of the particles volume fraction $\varphi_{p}$ and effect of the diffusivity $D_{i}$ and thickness $e_{i}$ of the interphase.

Considering the same interphase thickness and diffusivity for each particle volume fraction would not be the right hypothesis to represent the increase in relative permeability in the composite as a function of the particles volume fraction (Figure 12). According to Figure 12, the two-phase model would be enough for $\varphi_{p}=5.14\;\%v/v$ for a good model fitting of experimental relative permeability value. Considering an interphase of 2.5 µm and $D_{i}=1\times 10^{-10}\;m^{2}\cdot s^{-1}$ would slightly improve prediction for $\varphi_{p}=11.4\;\%v/v$ even if the prediction of the two-phase model was enough and included in the experimental variability of results obtained at this volume fraction. On the contrary, it would be absolutely necessary to consider an interphase of 5 µm (with $D_{i}=1\times 10^{-10}\;m^{2}\cdot s^{-1}$) for the $\varphi_{p}=19.52\;\%v/v$. However, reliability of this hypothesis still needs to be experimentally validated.

To sum up, the presence of an interphase layer around the inclusions, globally more diffusive than the particles could explain the high upturn of permeability curve for high particle volume fraction ($\varphi_{p}=19.52\;\%v/v$). A minimal interphase thickness would be required to form a continuous diffusion paths, which may occur between overlapping interphases.

## 4. Conclusions

In this paper, a 3D three-phase model was developed in order to predict water vapor permeability in micro-composite (PHBV matrix/WSF particles). This 3D study was essential to improve understanding of structure/material transfer relationships in bio-composite materials containing permeable particles. The developed two-phase model led to good prediction of the experimental relative permeability for the composites of $\varphi_{p}=5.14\;\%v/v$ and $\varphi_{p}=11.4\;\%v/v$. Nevertheless, for the composite of $\varphi_{p}=19.52\;\%v/v$, the results revealed that the mass transfer properties cannot be predicted directly from the mass transfer properties of each phase (matrix and particle) considering a binary system. Among the different hypothesis tested to explain the gap between model prediction and experimental results, an increase of matrix diffusivity (×3) in the composite with 19.52 %v/v of volume fraction would lead to a good model fitting. Alternatively, presence of an interphase of 5 µm between matrix and particles with its own diffusivity $1\times 10^{-10}\;m^{2}\cdot s^{-1}$ would permit to perfectly fit the experimental data for the highest volume fraction. Thorough analysis of published literature on PHBV based composites has permitted to conclude that probably both phenomena concomitantly occur. Results of this work highlighted that presence or not of an interphase, and thus choice of the model to be used would be strongly dependent on the particle volume fraction and its size distribution. This plenty justified the choice of developing a 3D, three-phase model based on experimental particles’ size distribution to well decipher the determinants of permeability change in composite of high volume fraction. In addition, such approach has permitted to multiply the number of computed structures and thus, to take into consideration the variability of the material structure leading to a better characterization and prediction of the material properties. The 3D FEM model proposed here was necessary to capture all the complexity of the bio-composites studied and to better construe impact of interphase, matrix and particle properties on macroscopic water vapor permeability.

## Data Availability

Raw simulation data used in the article could be uploaded from the publicly available dataset: https://doi.org/10.15454/D0GZDQ, accessed on 7 July 2021.

## Supplementary Materials

The following are available online at https://www.mdpi.com/article/10.3390/polym13142257/s1. Figure S1. Numerical process to generate the 3D microstructure of the RVE performed in MATLAB. Figure S2. Comparison between experimental relative permeability *P*/*P_m_* with calculated ones by using Maxwell-Wagner-Sillar equation. Figure S3. Evolution of the numerical relative permeability of water vapor as a function of the mesh element size for the composite of *φ_p_* = 5.14 %v/v. Mesh tests are performed with 10 structures. Figure S4. Evolution of the numerical relative permeability of water vapor as a function of the mesh element size for the composite of *φ_p_* = 11.4 %v/v. Mesh tests are performed with 10 structures. Figure S5. Evolution of the numerical relative permeability of water vapor as a function of the mesh element size for the composite of *φ_p_* = 19.52 %v/v. Mesh tests are performed with 8 structures. Numerical results corresponding to some mesh element sizes are not available due to mesh errors encountered on some simulations. Figure S6. Evolution of the numerical relative permeability of water vapor as a function of the diffusivity and thickness of the interphase for the composite of *φ_p_* = 5.14 %v/v: Comparison between experimental and numerical results. The numerical results (bullets) correspond to the average of the relative permeability of 20 structures (*e_i_* = 1 µm), 23 structures (*e_i_* = 2.5 µm) and 26 structures (*e_i_* = 5 µm). The volume fraction of the interphase was *φ_i_* = 1.85 ± 0.34 %v/v for *e_i_* = 1 µm, *φ_i_* = 5.71 ± 1.24 %v/v for *e_i_* = 2.5 µm and *φ_i_* = 15.19 ± 3.36 %v/v for *e_i_* = 5 µm. Figure S7. Evolution of the numerical relative permeability of water vapor as a function of the diffusivity and thickness of the interphase for the composite of *φ_p_* = 11.4 %v/v. The numerical results (bullets) correspond to the average of the relative permeability of 36 structures (*e_i_* = 1 µm), 36 structures (*e_i_* = 2.5 µm) and 21 structures (*e_i_* = 5 µm). The volume fraction of the interphase was *φ_i_* = 3.32 ± 0.9 %v/v for *e_i_* = 1 µm, *φ_i_* = 9.93 ± 2.69 %v/v for *e_i_* = 2.5 µm, and *φ_i_* = 22.47 ± 6.65 %v/v for *e_i_* = 5 µm. Figure S8. Evolution of the numerical relative permeability of water vapor as a function of the diffusivity of the interphase for the composite of *φ_p_* = 19.52 %v/v and *e_i_* = 1 µm: Comparison between experimental and numerical results. The numerical results corresponded to the relative permeability of 14 structures. *φ_i_* and np are the interphase volume fraction and the number of particles respectively. Figure S9. Evolution of the numerical relative permeability of water vapor as a function of the diffusivity of the interphase for the composite of *φ_p_* = 19.52 %v/v and *e_i_* = 2.5 µm: Comparison between experimental and numerical results. The numerical results corresponded to the relative permeability of 19 structures. *φ_i_* and np are the interphase volume fraction and the number of particles respectively. Figure S10. Evolution of the numerical relative permeability of water vapor as a function of the diffusivity of the interphase for the composite of *φ_p_* = 19.52 %v/v and *e_i_* = 5 µm: Comparison between experimental and numerical results. The numerical results corresponded to the relative permeability of 6 structures. *φ_i_* and np are the interphase volume fraction and the number of particles respectively.

## Nomenclature

Abbreviations	
RVE	Representative Volume Element
FEM	Finite Element Method
PHBV	Poly(3-HydroxyButyrate-*co*-3-HydroxyValerate)
WSF	Wheat Straw Fibers
2D, 3D	Two and Three Dimension
E	Mean
SD	Standard Deviation
PDF	Probability Density Function
CDF	Cumulative Distribution Function
*cte*	Constant value
Latin symbols	
$L_{x},L_{y},L_{z}$	RVE length along *x*-axis, *y*-axis and *z*-axis $\left(m\right)$
$a_{p},b_{p},c_{p}$	Major, minor and third axis of the particle $\left(m\right)$
$x_{p},y_{p},z_{p}$	Center coordinates of the particle $\left(m\right)$
$D_{k}$	Diffusivity of water vapor in the phase k $\left(m^{2}\cdot s^{-1}\right)$
$P_{k}$	Permeability of water vapor in the phase k $\left(\mathrm{mol}.m.m^{-2}\cdot s^{-1}\cdot {\mathrm{Pa}}^{-1}\right)$
P	Permeability of water vapor in the composite $\left(\mathrm{mol}\cdot m\cdot m^{-2}\cdot s^{-1}\cdot {\mathrm{Pa}}^{-1}\right)$
$c_{k}$	Concentration of water vapor in the phase k $\left(\mathrm{mol}\cdot m^{-3}\right)$
${\vec{J}}_{k}$	Molar surface flux vector of water vapor in the phase k $\left(\mathrm{mol}\cdot m^{-2}\cdot s^{-1}\right)$
$p_{upper}-p_{lower}$	Water vapor pressure differential across the film $\left(\mathrm{Pa}\right)$
K	Partition coefficient: concentration ratio between particles and matrix at equilibrium $\left(c_{p}/c_{m}\right)$ (-)
M	Velocity (non-physical property) $\left(m\cdot s^{-1}\right)$
$e_{i}$	Interphase thickness $\left(m\right)$
$n_{p}$	Number of particles (-)
Greek symbols	
$\alpha_{p}$	Aspect ratio: ratio between major and minor axis of the particle ($a_{p}/b_{p})$ (-)
$\varphi_{k}$	Volume fraction of the phase k: ratio between the volume of the phase k and the composite volume $\left(\%v/v\right)$
$\phi_{z}$	Molar flux (along *z*-axis) of water vapor across a composite face $\left(\mathrm{mol}\cdot s^{-1}\right)$
$\theta_{p1}$	Azimuth angle: angle between the *x*-axis and the orthogonal projection of the semi-major axis onto the xy-plane (degree°)
$\theta_{p2}$	Elevation angle: angle between the semi-major axis and its orthogonal projection onto the xy-plane (degree°)
Subscripts	
m	Matrix
p	Particle
i	Interphase

## Appendix A

Probability Density Function (PDF) of log-normal distribution $f\left(x\right)$ of random variable *x* with parameters μ and σ was defined as

(A1) $$f\left(x\right)=\frac{1}{x\sigma \sqrt{2\pi }}e^{-\frac{{\left(ln\left(x\right)-\mu \right)}^{2}}{2\sigma^{2}}}\;x\in ]0+\infty [$$

The mean E, variance V and standard deviation SD of log-normal distribution were:

(A2) $$E=e^{\mu +\frac{\sigma^{2}}{2}}\;;\;V=\left(e^{\sigma^{2}}-1\right)e^{2µ+\sigma^{2}};\;SD=\sqrt{V}$$

PDF of truncated log-normal distribution $g\left(x\right)$ of random variable *x* with parameters μ, σ, $x_{min}$ and $x_{max}$ was defined as:

(A3) $$g\left(x\right)=\frac{f\left(x\right)}{\Phi \left(\frac{ln\left(x_{min}\right)-\mu }{\sigma }\right)-\Phi \left(\frac{ln\left(x_{max}\right)-\mu }{\sigma }\right)}\;x\in \left[x_{min},x_{max}\right]$$

where $f\left(x\right)$ is the PDF of log-normal distribution and $\Phi \left(x\right)=\frac{1}{\sqrt{2\pi }}\int_{-\infty }^{x}e^{-x^{2}/2}dx$ is the Cumulative Distribution Function (CDF) of the standard normal distribution. The mean $E_{trunc}$, variance $V_{trunc}$ and standard deviation ${\mathrm{SD}}_{trunc}$ of truncated log-normal distribution were:

(A4) $$E_{\;trunc}=E\frac{T_{2}}{T_{1}}$$

(A5) $${V\;}_{trunc}=E^{2}\left(\frac{T_{3}}{T_{1}}-\frac{T_{2}^{2}}{T_{1}^{2}}\right)+V\frac{T_{3}}{T_{1}}$$

(A6) $${SD\;}_{trunc}=\sqrt{{V\;}_{trunc}}$$

with

(A7) $$T_{1}=\Phi \left(\frac{ln\left(x_{max}\right)-\mu }{\sigma }\right)-\Phi \left(\frac{ln\left(x_{min}\right)-\mu }{\sigma }\right)\;$$

(A8) $$T_{2}=\Phi \left(\frac{ln\left(x_{max}\right)-\mu }{\sigma }\;-\sigma \right)-\Phi \left(\frac{ln\left(x_{min}\right)-\mu }{\sigma }\;-\sigma \right)$$

(A9) $$T_{3}=\Phi \left(\frac{ln\left(x_{max}\right)-\mu }{\sigma }\;-2\sigma \right)-\Phi \left(\frac{ln\left(x_{min}\right)-\mu }{\sigma }\;-2\sigma \right)$$
