# Supplementary material for: 3D Modelling of Mass Transfer into Bio-Composite
Source: Polymers (Basel). 2021 Jul 9;13(14):2257. doi: 10.3390/polym13142257 (PMC8309300; doi:10.3390/polym13142257)
Supplement: Supplementary file 1 [file polymers-13-02257-s001.zip › polymers-1274056-supplementary.pdf]

polymers

MDPI

Supplementary material

3D Modelling of Mass Transfer into Bio-Composite

Marouane Kabbej, Valérie Guillard *, Hélène Angellier-Coussy, Caroline Wolf, Nathalie Gontard and Sébastien Gaucel

IATE, Univ Montpellier, CIRAD, INRAE, Institut Agro, Montpellier 34060, France; marouane.kabbej@umontpellier.fr (M.K.); helene.coussy@umontpellier.fr (H.A.-C.); wolf.caroline1@gmail.com (C.W.); nathalie.gontard@inrae.fr (N.G.); sebastien.gaucel@inrae.fr (S.G.)

* Correspondence: valerie.guillard@umontpellier.fr

Figure S1: Numerical process to generate the 3D microstructure of the RVE performed in MATLAB 2

Figure S2: Comparison between experimental relative permeability P/Pm with calculated ones by using Maxwell-Wagner-Sillar equation 3

Figure S3. Evolution of the numerical relative permeability of water vapor as a function of the mesh element size for the composite of $\varphi p = 5.14\ \%v/v$. Mesh tests are performed with 10 structures. 4

Figure S4. Evolution of the numerical relative permeability of water vapor as a function of the mesh element size for the composite of $\varphi p = 11.4\ \%v/v$. Mesh tests are performed with 10 structures. 4

Figure S5. Evolution of the numerical relative permeability of water vapor as a function of the mesh element size for the composite of $\varphi p = 19.52\ \%v/v$. Mesh tests are performed with 8 structures. Numerical results corresponding to some mesh element sizes are not available due to mesh errors encountered on some simulations. 5

Figure S6. Evolution of the numerical relative permeability of water vapor as a function of the diffusivity and thickness of the interphase for the composite of $\varphi p = 5.14\ \%v/v$: Comparison between experimental and numerical results. The numerical results (bullets) correspond to the average of the relative permeabity of 20 structures $(ei = 1\ \mu m)$, 23 structures $(ei = 2.5\ \mu m)$ and 26 structures $(ei = 5\ \mu m)$. The volume fraction of the interphase was $\varphi i = 1.85 \pm 0.34\ \%v/v$ for $ei = 1\ \mu m$, $\varphi i = 5.71\ \pm 1.24\ \%v/v$ for $ei = 2.5\ \mu m$ and $\varphi i = 15.19\ \pm 3.36\ \%v/v$ for $ei = 5\ \mu m$. 6

Figure S7. Evolution of the numerical relative permeability of water vapor as a function of the diffusivity and thickness of the interphase for the composite of $\varphi p = 11.4\ \%v/v$. The numerical results (bullets) correspond to the average of the relative permeabity of 36 structures $(ei = 1\ \mu m)$, 36 structures $(ei = 2.5\ \mu m)$ and 21 structures $(ei = 5\ \mu m)$. The volume fraction of the interphase was $\varphi i = 3.32 \pm 0.9\ \%v/v$ for $ei = 1\ \mu m$, $\varphi i = 9.93\ \pm 2.69\ \%v/v$ for $ei = 2.5\ \mu m$, and $\varphi i = 22.47\ \pm 6.65\ \%v/v$ for $ei = 5\ \mu m$. 6

Figure S8. Evolution of the numerical relative permeability of water vapor as a function of the diffusivity of the interphase for the composite of $\varphi p = 19.52\ \%v/v$ and $ei = 1\ \mu m$: Comparison between experimental and numerical results. The numerical results corresponded to the relative permeabity of 14 structures. φi and np are the interphase volume fraction and the number of particles respectively. 7

Figure S9. Evolution of the numerical relative permeability of water vapor as a function of the diffusivity of the interphase for the composite of $\varphi p = 19.52\ \%v/v$ and $ei = 2.5\ \mu m$: Comparison between experimental and numerical results. The numerical results corresponded to the relative permeability of 19 structures. φi and np are the interphase volume fraction and the number of particles respectively. 7

Figure S10. Evolution of the numerical relative permeability of water vapor as a function of the diffusivity of the interphase for the composite of $\varphi p = 19.52\ \%v/v$ and $ei = 5\ \mu m$: Comparison between experimental and numerical results. The numerical results corresponded to the relative permeabity of 6 structures. φi and np are the interphase volume fraction and the number of particles respectively. 8

S1. Organizational chart summarizing the 3D structure generation algorithm

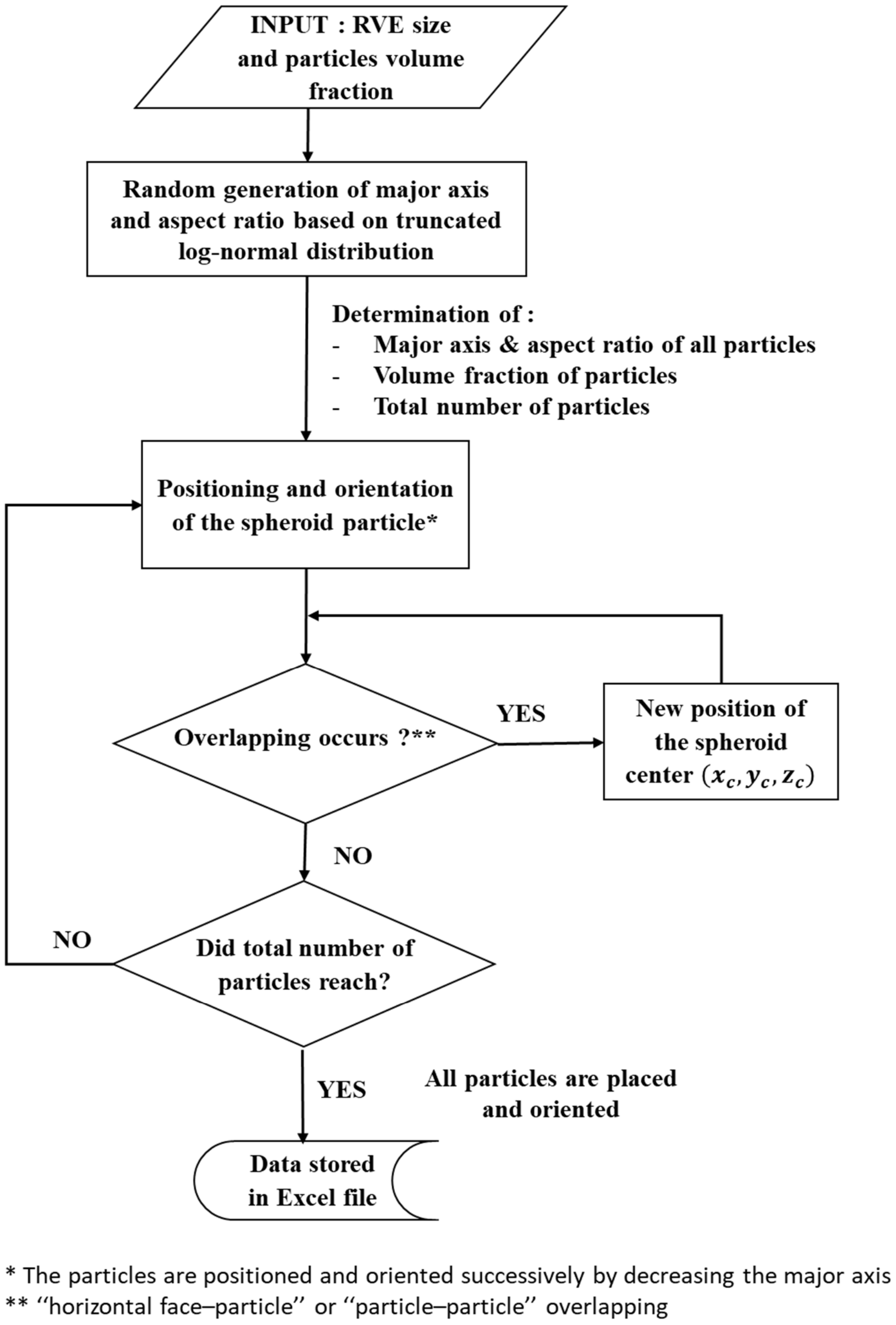

Figure S1. Numerical process to generate the 3D microstructure of the RVE performed in MATLAB.

S2. Adjustment of analytical Maxwell-Wagner-Sillar equation onto experimental permeability results obtained on made of PHBV as continuous phase and WSF as dispersed and permeable phase

The analytic model of Maxwell-Wagner-Sillars Eq. (1) relates the composite permeability P to the matrix permeability P_m, the particle permeability P_p, the particle volume fraction φ_p and a shape factor n. It is applicable to diluted ellipsoid dispersions.

$$\frac{P}{P_m} = \frac{nP_p+(1-n)P_m+(1-n)(P_p-P_m)\varphi_p}{nP_p+(1-n)P_m-n(P_p-P_m)\varphi_p} \quad (1)$$

Eq. (1) was fitted to the experimental data showing the evolution of the ratio P/P_m as a function of fibre particles volume fraction. The value of the input particle permeability was calculated as the product of experimental particle diffusivity by experimental particle solubility obtained from dynamic sorption experiments [1], $P_p = 1664 \pm 451 \times 10^{-13}\ \mathrm{mol.m.m^{-2} \cdot s^{-1} \cdot Pa^{-1}}$.

The resulting fitting value of parameter $n = 0.044$ correspond to the case of particles representing prolate ellipsoids $(0 \leq n \leq 1/3)$, i.e. the longest axis of the ellipsoid is directed along the flux direction.

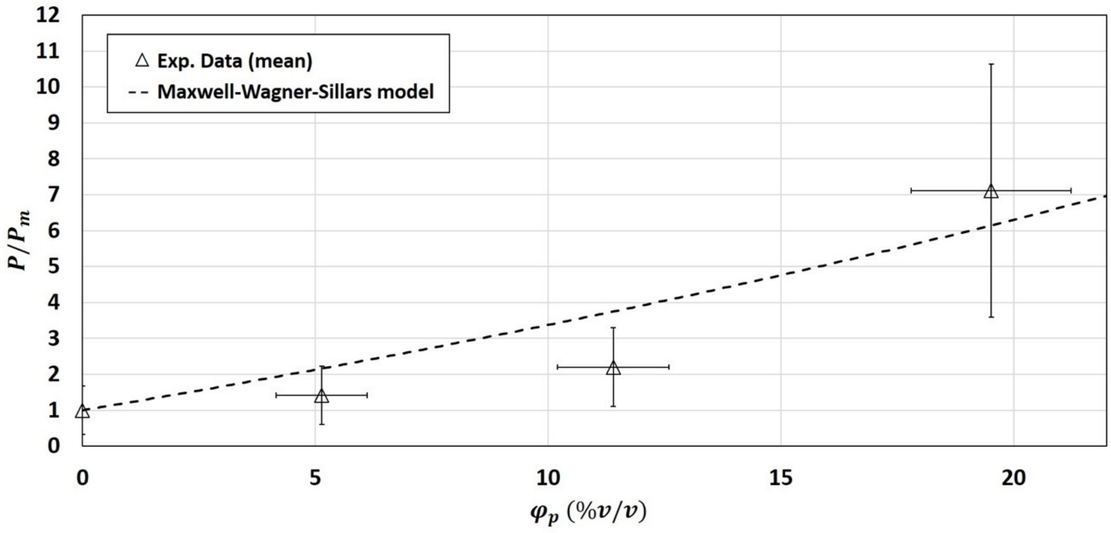

Figure S2. Comparison between experimental relative permeability P/P_m with calculated ones by using Maxwell-Wagner-Sillar equation

1. [1] Wolf C, Guillard V, Angellier-Coussy H, Silva GGD, Gontard N. Water vapor sorption and diffusion in wheat straw particles and their impact on the mass transfer properties of biocomposites. J Appl Polym Sci 2016;133:1–10. https://doi.org/10.1002/app.43329.

S3. Evolution of the numerical relative permeability of water vapor as a function of the mesh element size for the two-phase model

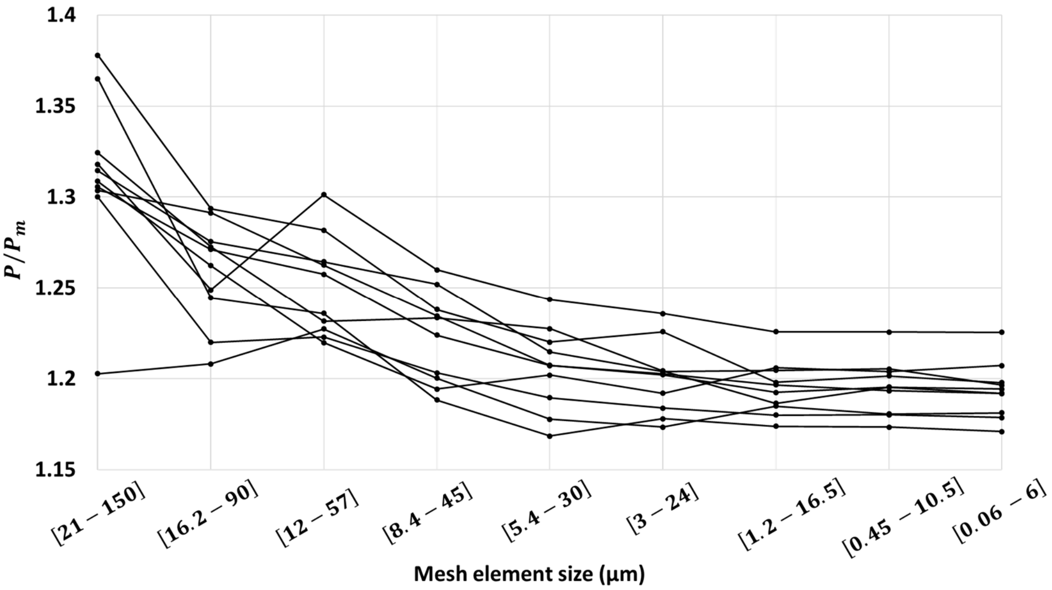

Figure S3. Evolution of the numerical relative permeability of water vapor as a function of the mesh element size for the composite of $\varphi_p = 5.14\ \%v/v$. Mesh tests are performed with 10 structures.

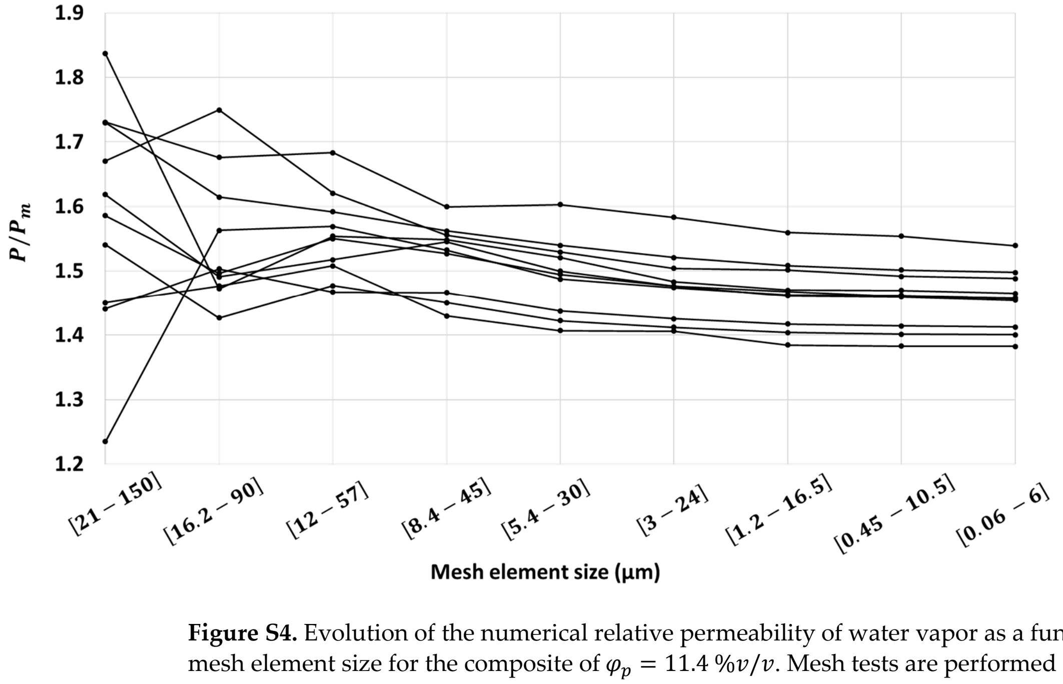

Figure S4. Evolution of the numerical relative permeability of water vapor as a function of the mesh element size for the composite of $\varphi_p = 11.4\ \%v/v$. Mesh tests are performed with 10 structures.

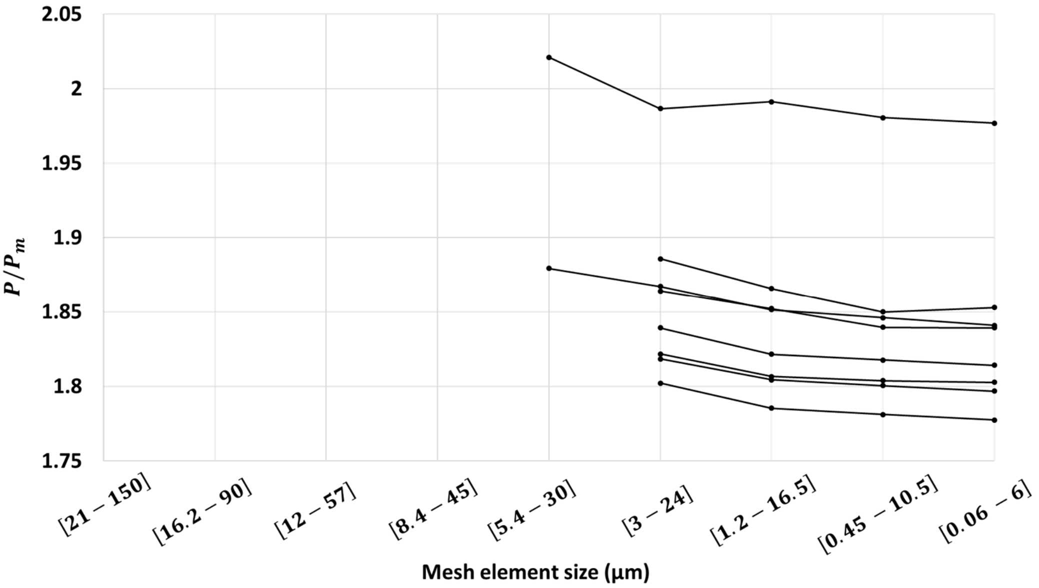

Figure S5. Evolution of the numerical relative permeability of water vapor as a function of the mesh element size for the composite of $\varphi_p = 19.52\ \%v/v$. Mesh tests are performed with 8 structures. Numerical results corresponding to some mesh element sizes are not available due to mesh errors encountered on some simulations.

S4. Evolution of the numerical relative permeability of water vapor as a function of the diffusivity of the interphase

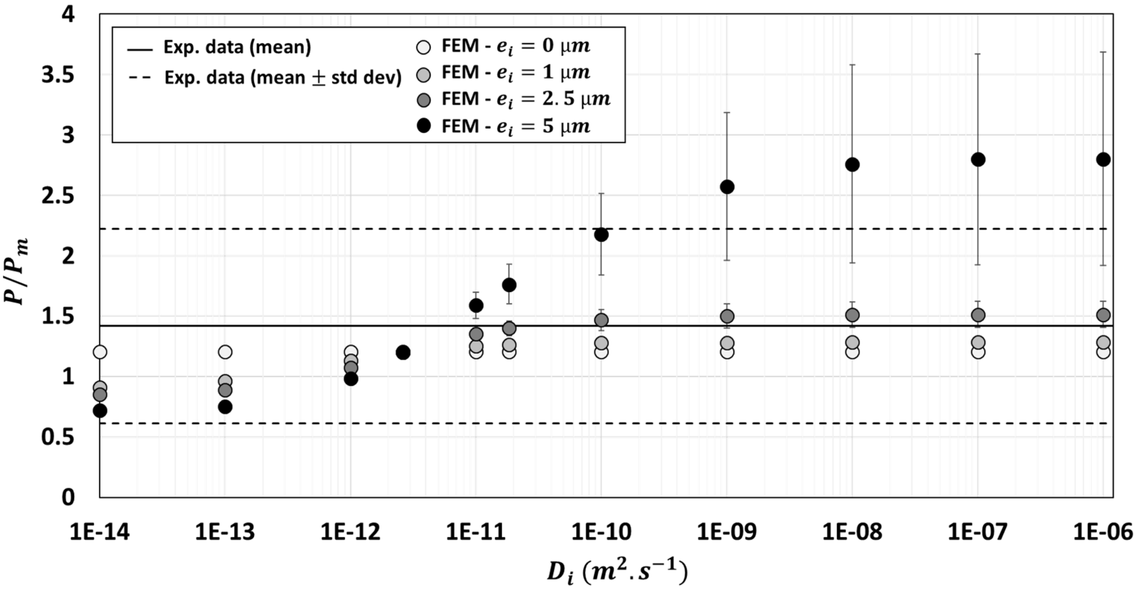

Figure S6. Evolution of the numerical relative permeability of water vapor as a function of the diffusivity and thickness of the interphase for the composite of $\varphi_p = 5.14\ \%v/v$: Comparison between experimental and numerical results. The numerical results (bullets) correspond to the average of the relative permeabity of 20 structures ($e_i = 1$ µm), 23 structures ($e_i = 2.5$ µm) and 26 structures ($e_i = 5$ µm). The volume fraction of the interphase was $\varphi_i = 1.85 \pm 0.34\ \%v/v$ for $e_i = 1$ µm, $\varphi_i = 5.71 \pm 1.24\ \%v/v$ for $e_i = 2.5$ µm and $\varphi_i = 15.19 \pm 3.36\ \%v/v$ for $e_i = 5$ µm.

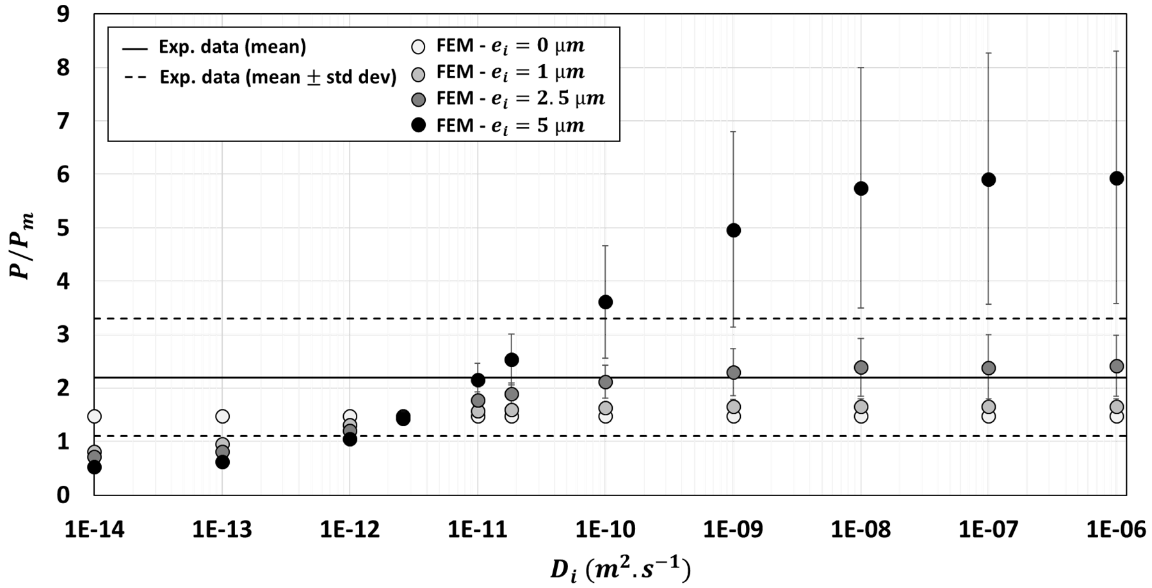

Figure S7. Evolution of the numerical relative permeability of water vapor as a function of the diffusivity and thickness of the interphase for the composite of $\varphi_p = 11.4\ \%v/v$. The numerical results (bullets) correspond to the average of the relative permeabity of 36 structures ($e_i = 1$ µm), 36 structures ($e_i = 2.5$ µm) and 21 structures ($e_i = 5$ µm). The volume fraction of the interphase was $\varphi_i = 3.32 \pm 0.9\ \%v/v$ for $e_i = 1$ µm, $\varphi_i = 9.93 \pm 2.69\ \%v/v$ for $e_i = 2.5$ µm, and $\varphi_i = 22.47 \pm 6.65\ \%v/v$ for $e_i = 5$ µm.

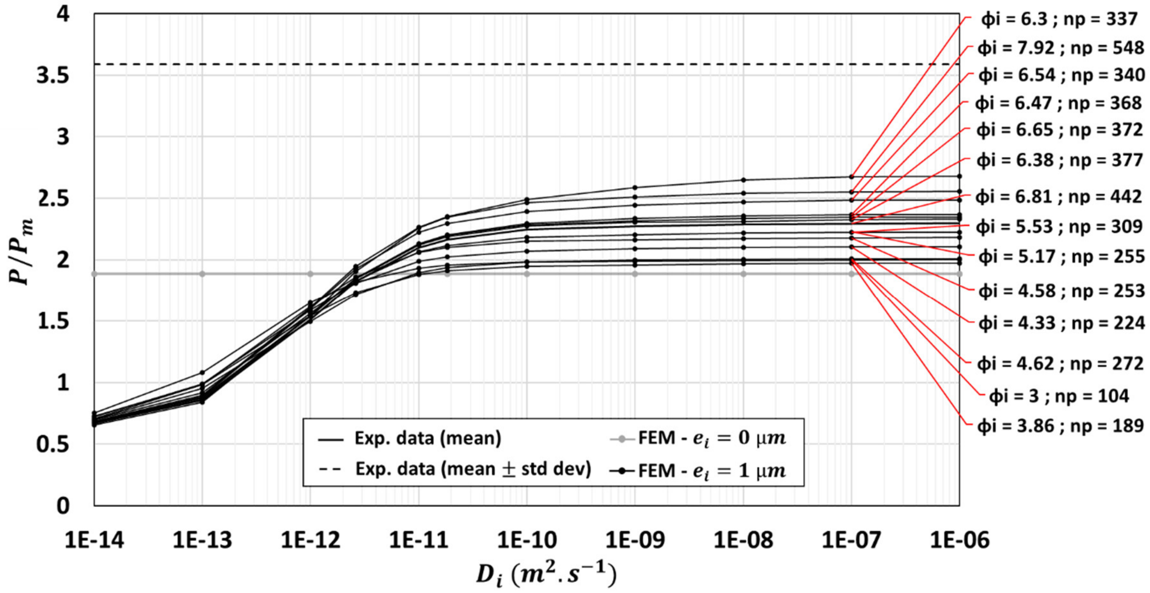

Figure S8. Evolution of the numerical relative permeability of water vapor as a function of the diffusivity of the interphase for the composite of $\varphi_p = 19.52\ \%v/v$ and $e_i = 1\ \mu m$: Comparison between experimental and numerical results. The numerical results corresponded to the relative permeabity of 14 structures. φ_i and n_p are the interphase volume fraction and the number of particles respectively.

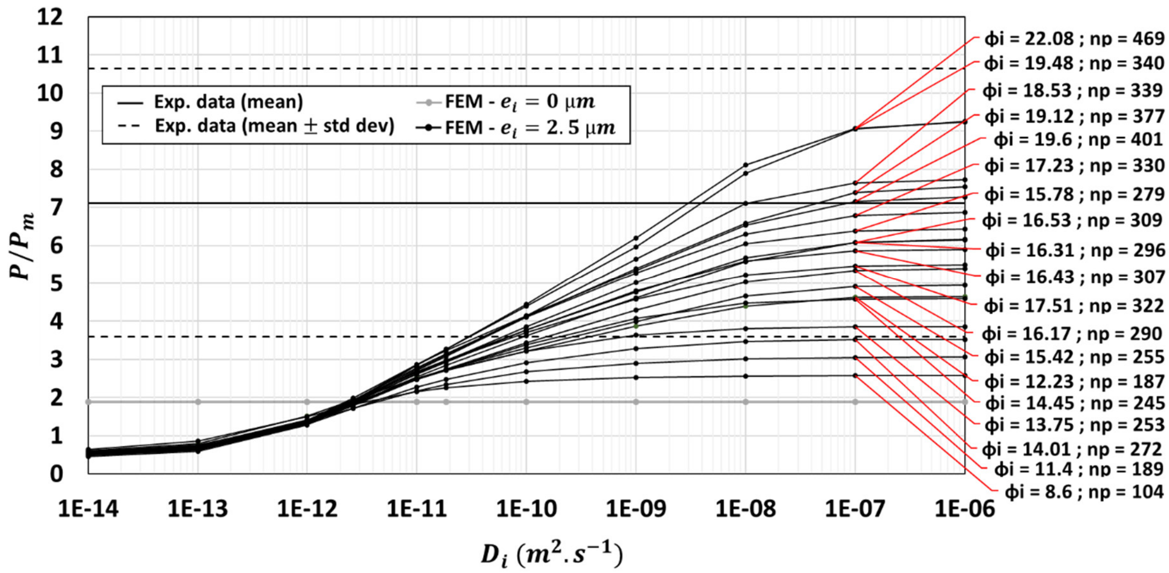

Figure S9. Evolution of the numerical relative permeability of water vapor as a function of the diffusivity of the interphase for the composite of $\varphi_p = 19.52\ \%v/v$ and $e_i = 2.5\ \mu m$: Comparison between experimental and numerical results. The numerical results corresponded to the relative permeability of 19 structures. φ_i and n_p are the interphase volume fraction and the number of particles respectively.

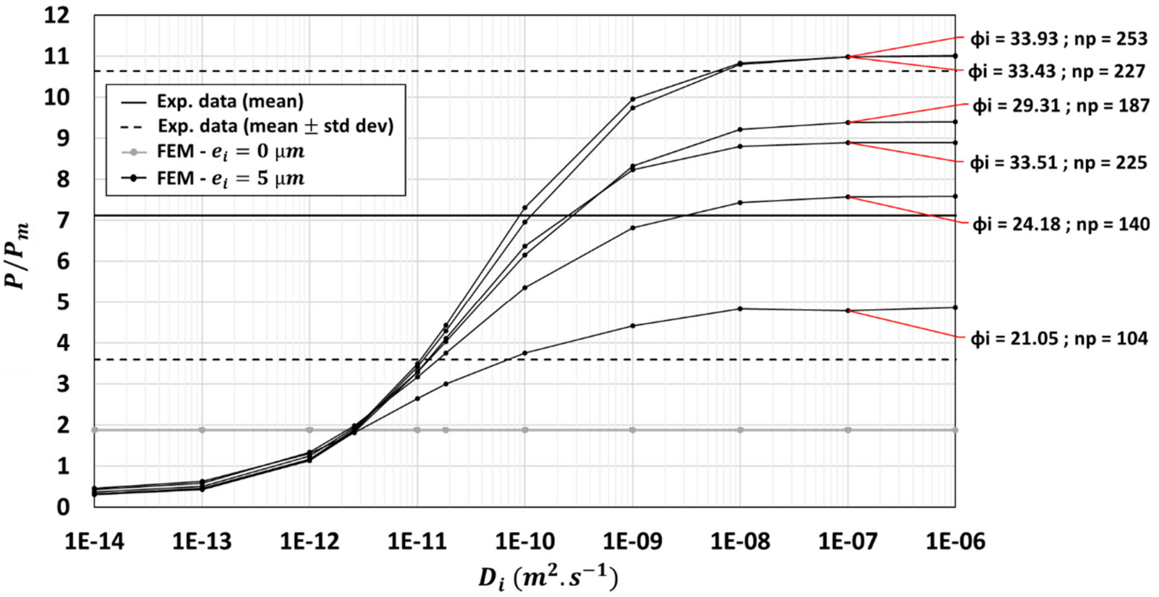

Figure S10. Evolution of the numerical relative permeability of water vapor as a function of the diffusivity of the interphase for the composite of $\varphi_p = 19.52\ \%v/v$ and $e_i = 5\ \mu m$: Comparison between experimental and numerical results. The numerical results corresponded to the relative permeabity of 6 structures. φ_i and n_p are the interphase volume fraction and the number of particles respectively.
